# Internal validation of a residual thyroid recurrence model after PTC

**DOI:** 10.3389/fonc.2026.1827099

**Published:** 2026-07-31

**Authors:** Ruohan Shi, Lisen Zhu, Jia Liu, Fuzhi Zhu, Weijia Wang, Gongsheng Jin

**Affiliations:** Department of the First Affiliated Hospital of Bengbu Medical University, Bengbu, China

**Keywords:** clinical utility, logistic regression model, papillary thyroid carcinoma, prediction model, residual thyroid recurrence, risk factors

## Abstract

**Background:**

At present, the risk factors contributing to the recurrence of papillary thyroid carcinoma (PTC) in residual thyroid tissue post-surgery are not yet fully understood. This study sought to identify multivariable predictors (MVPs) associated with such recurrence and to develop a dynamic, individualized nomogram. The objective is to establish a stratification framework to inform precision “risk-intervention” decision-making for patients at elevated risk of recurrence.

**Methods:**

A cohort of 2,512 patients diagnosed with thyroid cancer and treated at the First Affiliated Hospital of Bengbu Medical University between January 2014 and December 2024 was analyzed. The cohort was stratified into a training set (n = 1,760) and a validation set (n = 752) using stratified random sampling with a 7:3 ratio. To address the class imbalance between recurrent and non-recurrent cases in the residual thyroid, the Random Over-Sampling Examples (ROSE) method was employed on the training set. Variable selection was conducted using univariate and multivariate logistic regression, LASSO regression, and standard stepwise regression based on the Akaike Information Criterion (AIC). A nomogram was subsequently developed by integrating the coefficients of the selected variables from the logistic regression model. The performance of the nomogram was assessed in both cohorts in terms of discrimination, calibration, and clinical utility.

**Results:**

In a study of 2,512 thyroid cancer patients who received primary treatment, 146 experienced recurrence in the remaining thyroid tissue. Using univariate and multivariate logistic regression and LASSO regression for feature selection, four independent predictors were identified for the final predictive model: maximum tumor diameter, number of metastatic lymph nodes, lymph node positivity rate, and age. The nomogram showed strong discriminatory ability, with an AUC of 0.7518 in the training set and 0.8347 in the validation set. It also demonstrated excellent calibration and clinical utility across all groups. The best cut-off value for the nomogram score to identify patients with residual thyroid recurrence was 0.245, with scores above this linked to a significantly worse postoperative outcome.

**Conclusions:**

We have developed and validated an innovative clinical prediction model specifically designed to assess the risk of recurrence in residual thyroid tissue. This model serves as a valuable tool for clinicians, facilitating personalized decision-making and representing a significant advancement in the postoperative management of papillary thyroid carcinoma.

## 1 Introduction

Thyroid cancer represents the most common endocrine malignancy globally, with its incidence demonstrating a marked increase in recent years. Among the various pathological subtypes, papillary thyroid carcinoma (PTC) is the most prevalent, constituting 80% to 90% of all thyroid cancer cases. Despite the generally favorable prognosis associated with PTC, accurate risk stratification and the dynamic prediction of long-term recurrence—particularly within the residual thyroid tissue or cervical lymph nodes post-thyroidectomy—continue to pose substantial challenges in clinical practice (1, 2).

At present, both international and domestic guidelines predominantly depend on the initial risk stratification system established by the American Thyroid Association (ATA), which is further supported by the monitoring of serum stimulated thyroglobulin (sTg) levels and the use of neck ultrasonography (3). This framework is a static classification relying on immediate postoperative pathology, and it inherently struggles to incorporate evolving factors like changes in sTg levels, interference from TgAb, and variations in treatment response over time (4). More significantly, current systems lack a dedicated risk assessment for “recurrence in the residual thyroid,” a distinct clinical event (5). As a result, certain patients categorized as “low risk” may nonetheless encounter delayed recurrence, whereas numerous “intermediate-risk” patients may be subjected to overly intensive follow-up protocols, leading to unwarranted psychological stress and increased medical expenses (6). This highlights a critical issue in clinical practice, wherein the “one-size-fits-all” management approach results in an inequitable distribution of medical resources and inadequately addresses the specific needs of individual patients.

Consequently, the development of a predictive tool that can dynamically and quantitatively evaluate the risk of recurrence in the residual thyroid is of substantial importance for the realization of individualized and precise postoperative follow-up management. This study seeks to address these gaps: (1) lack of a dedicated tool for residual thyroid recurrence; (2) static nature of current ATA stratification; and (3) absence of individualized probability prediction. To fill these gaps, we developed and internally validated a clinical prediction model using routine clinicopathological variables and providing individualized risk via a nomogram. The primary innovation of this research is the isolation of “recurrence in the residual thyroid”—a pivotal local event—from the broader notion of “recurrence,” thereby establishing it as an independent research endpoint. Utilizing objective and precisely quantifiable core variables, we have developed and internally validated a specialized clinical prediction model designed to achieve a nuanced gradient differentiation of recurrence risk (7).Recurrence in the residual thyroid is clinically distinct from nodal or distant recurrence. The former can be managed by completion thyroidectomy or local ablation, whereas the latter often require systemic therapy. Therefore, focusing on this specific endpoint enables tailored risk stratification and intervention.

This study focuses on predicting long-term recurrence risk (3–5 years) in adult patients with residual thyroid tissue after PTC surgery, assessed within 3–6 months post-surgery. It targets surgical oncologists, endocrinologists, and nuclear medicine specialists, offering risk probabilities or levels to aid in personalized imaging follow-ups and surveillance strategies. The goal is to enhance postoperative thyroid cancer management, optimize resource use, and improve patient quality of life (8, 9).

## 2 Methods

### 2.1 Participants

This retrospective single-center study was conducted at the First Affiliated Hospital of Bengbu Medical University from January 2014 to December 2024. Inclusion criteria included: (1) pathologically confirmed unilateral papillary thyroid carcinoma (PTC); (2) less-than-total thyroidectomy with preserved thyroid tissue, irrespective of cervical lymph node dissection; (3) a systematic follow-up of 3 to 5 years in line with clinical guidelines; (4) comprehensive clinical records, including preoperative tests, imaging, surgical and pathological reports, and postoperative adjuvant therapy records; (5) regular postoperative ultrasonography and thyroid function tests. Exclusion criteria included: (1) Reoperation cases; (2) Non-papillary thyroid carcinoma types or other malignancies; (3) Preoperative or early postoperative distant metastasis or severe extrathyroidal extension; (4) Missing essential baseline data not fixable by imputation; (5) Incomplete follow-up or less than 5 years for non-recurrent patients; (6) Other primary malignancies or death unrelated to thyroid cancer. We used a validation strategy combining hold-out validation and Bootstrap resampling for this retrospective cohort. The Ethics Committee of the First Affiliated Hospital of Bengbu Medical University approved the study, and all participants or their guardians provided written informed consent (10).

### 2.2 Outcome

The primary outcome was a prognostic endpoint, characterized by the recurrence of disease in the residual thyroid tissue within a 3–5 year postoperative follow-up period, as confirmed through both imaging and pathological assessment. A comprehensive diagnostic criterion was employed as the reference standard: cytopathological results from ultrasound-guided fine-needle aspiration biopsy (FNA) constituted the core gold standard, augmented by positive findings from diagnostic radioiodine whole-body scintigraphy (Dx-WBS) and a progressive increase in serum thyroglobulin (Tg) levels (11). The interpretations were independently conducted by a minimum of two experienced thyroid specialists, comprising radiologists and pathologists, who were blinded to the patients’ additional predictive factors. This endpoint was chosen because it has direct clinical actionability: high-risk patients can receive intensified surveillance or early re-intervention, while low-risk patients avoid unnecessary procedures. Any discrepancies were addressed and resolved through multidisciplinary team (MDT) discussions until consensus was achieved (12).

### 2.3 Predictors and variable selection

Predictor variables were identified from postoperative clinical records and laboratory assessments, with the selection process being pre-established according to clinical accessibility and expert consensus (13). To address missing values, multiple imputation was employed, incorporating all candidate variables into the imputation model and executing it independently during the training phase. Specifically, predictive mean matching (PMM) was utilized for numerical variables, logistic regression (LOGREG) for binary factors, and polytomous logistic regression (POLYREG) for multicategorical factors. A fixed random seed was used to ensure(set.(seed123)) reproducibility.

To meet the scale needs of later modeling algorithms, all continuous predictors were standardized using Z-score normalization, converting them to a mean of 0 and a standard deviation of 1. To prevent data leakage, the standardization parameters were derived only from the training set and then applied to the validation and test sets. The transformation formula is as follows:


$$X_{scaled}=\frac{X-\mu_{train}}{\sigma_{train}}$$

**Figure 1:**
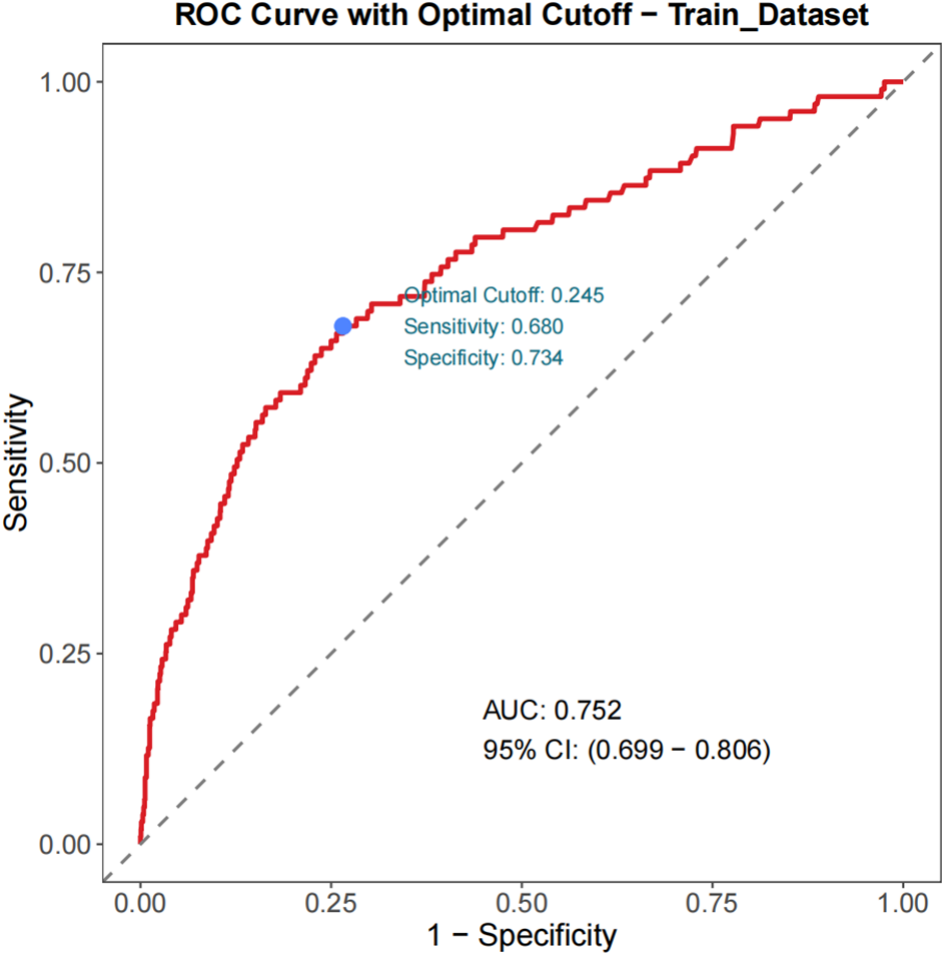
ROC curves of the prediction model. The figure shows the discriminatory performance of the nomogram for distinguishing patients with and without recurrence in the training and internal validation sets. The area under the curve (AUC) was 0.7518 (95% CI: 0.712–0.792) in the training set and 0.8347 (95% CI: 0.785–0.884) in the validation set, indicating good discriminative ability. The diagonal line represents the reference line (AUC = 0.5).

Where μtrain and σtrain represent the mean and standard deviation of the variable in the training set, respectively. This procedure aimed to eliminate dimensional effects and enhance the convergence speed and stability of the model, which is particularly critical for algorithms based on distance or regularization, such as LASSO. It is important to note that Z-score standardization was used only for model training (LASSO and logistic regression). For the final nomogram, all predictors were converted back to their original measurement scales to enhance clinical usability. Therefore, the values displayed in the nomogram (**Figure 1**) are all positive, whereas the standardized values used internally range from negative to positive. Categorical variables were automatically converted into factors. To mitigate the risk of multicollinearity and overfitting, the Least Absolute Shrinkage and Selection Operator (LASSO) regression was employed within a 10-fold cross-validation framework to identify the optimal subset of features. The regularization parameter λ was determined using the one-standard-error criterion from the cross-validation curve. The final features were ultimately established based on the training set (14).

### 2.4 Model development

A logistic regression algorithm was employed within the training set. The hyperparameters (regularization strength λ) were optimized through 10-fold cross-validation combined with a random search strategy, using the Area Under the Curve (AUC) as the primary evaluation metric. Given the initial ratio of recurrence to non-recurrence (146/2366≈1:16), the Random Over-Sampling Examples (ROSE) technique – a hybrid method that generates synthetic samples of the minority class (recurrence) using a smoothed bootstrap (kernel density estimation)and randomly under-samples the majority class – was applied to the training set, achieving a recurrence to non-recurrence ratio of approximately 1:3. Notably, the validation set retained its original distribution to ensure the clinical validity of the performance evaluation. Probability calibration was performed using the calibrate function from the rms package via Bootstrap internal validation. The optimal probability cut-off was determined by maximizing the Youden index (J = Sensitivity + Specificity – 1). For each possible threshold, sensitivity and specificity were calculated from the model-predicted probabilities, and the threshold yielding the highest J value was selected as the optimal cut-off. To facilitate clinical deployment, a logistic regression model incorporating the selected variables was constructed, with its predictive equation preserved and visualized as a nomogram (15).

### 2.5 Model evaluation

After training the model, its performance was assessed on an internal validation set using key metrics: discriminatory (AUC, sensitivity, specificity, accuracy, F1-score), calibration (calibration curves, intercept, slope, Brier score), and clinical utility (Decision Curve Analysis, DCA). Confidence intervals for all metrics were calculated via Bootstrap (1,000 resamples). AUCs between training and validation sets were compared using DeLong’s test for correlated ROC curves (DeLong et al., 1988). This test is appropriate because both AUCs derive from the same logistic regression model applied to different datasets, and no nested models were involved. Positive/negative predictive values and the confusion matrix were reported based on the Youden index threshold. The model’s net benefit was evaluated through DCA (16).

### 2.6 Statistical analysis

Statistical analyses were conducted with R software (version 4.5.1). Categorical variables are shown as frequencies (percentages) and compared using the chi-square test or Fisher’s exact test. Continuous variables are given as mean ± SD or median (IQR), with group comparisons made using the independent samples t-test or Mann–Whitney U test. All tests were two-sided with significance at p < 0.05, and the Benjamini–Hochberg correction was used for multiple comparisons when necessary.

The primary plotting and modeling tasks were conducted utilizing specialized R packages, such as tidyverse, caret, pROC, glmnet, rms, and ResourceSelection. The comprehensive analysis code, along with a minimally reproducible example, is available in the **Supplementary Material** accompanying this paper.

## 3 Results

### 3.1 Baseline characteristics

**Figure 2:**
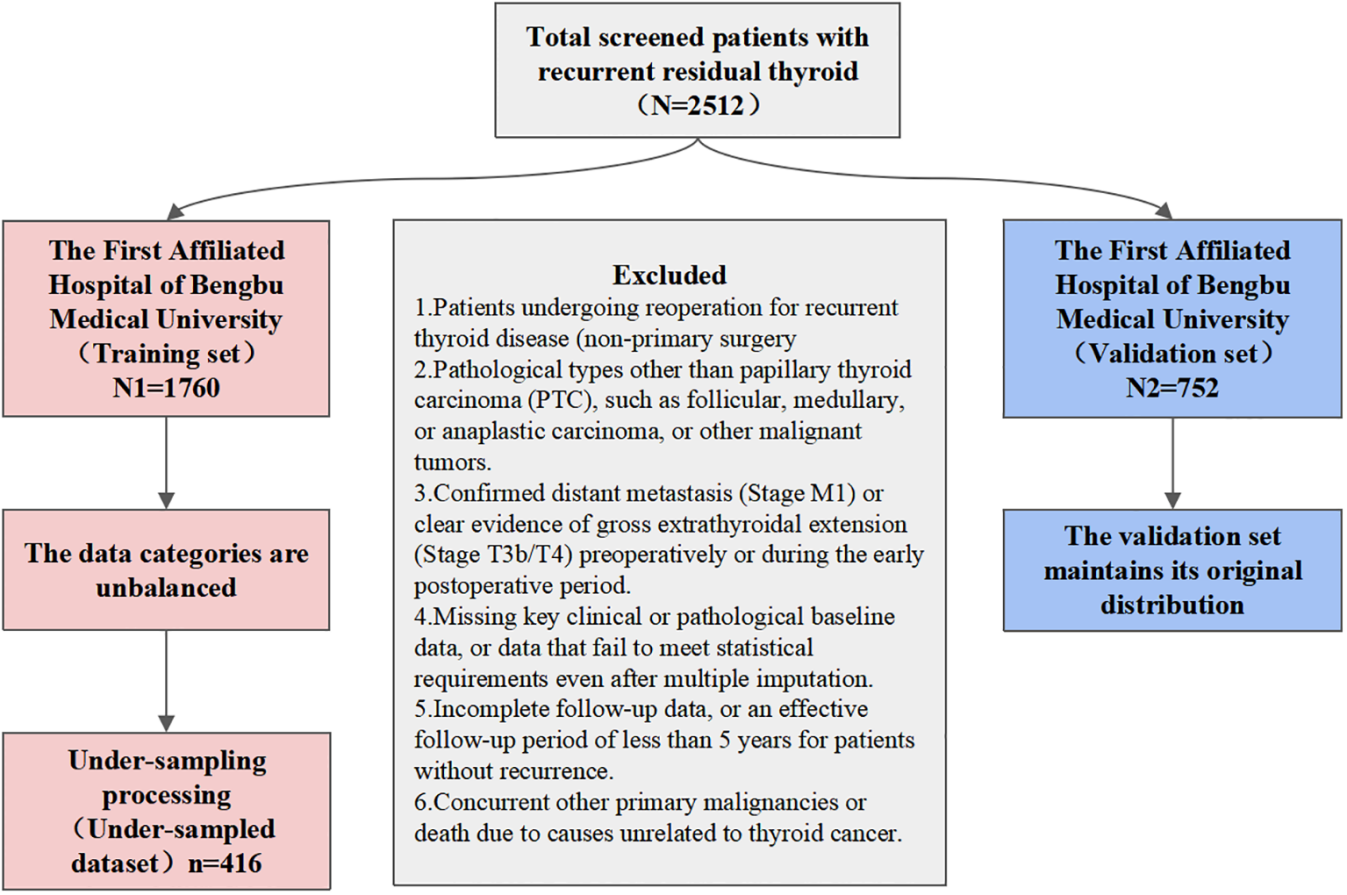
Flowchart of study participants in the training and validation cohorts.

The study included 2,512 subjects, split into a training set (N1) and an internal validation set (N2), as shown in **Figure 2**. Endpoints were based on a comprehensive reference standard, primarily using cytopathological results from ultrasound-guided fine-needle aspiration (FNA), along with positive diagnostic radioiodine whole-body scintigraphy (Dx-WBS) findings and rising serum thyroglobulin (Tg) levels (11). **Table 1** provides a summary of the baseline characteristics of the study population.

**Table 1:** General characteristics.

Variables	Total (n = 2512)	No Recurrence (n = 2366)	Recurrence (n = 146)	p	statistic
sex, n (%)				0.111	2.545
female	1981 (78.9)	1874 (79.2)	107 (73.3)		
male	531 (21.1)	492 (20.8)	39 (26.7)		
age, years Median (Q1,Q3)	46 (38, 54)	46 (38, 54)	47 (34, 56)	0.575	167948.5
Tumor type, n (%)				< 0.001	34.536
Micro Papillary Carcinoma	1238 (49.3)	1201 (50.8)	37 (25.3)		
Papillary Carcinoma	1274 (50.7)	1165 (49.2)	109 (74.7)		
tumor_max_diameter_cm, Median (Q1,Q3)	0.8 (0.5, 1.5)	0.8 (0.5, 1.3)	1.3 (0.7, 2.3)	< 0.001	113485.5
Lymph node status, n (%)				< 0.001	80.179
Negative	1542 (61.4)	1504 (63.6)	38 (26)		
Positive	970 (38.6)	862 (36.4)	108 (74)		
The number of lynph nodes, Median (Q1,Q3)	0 (0, 2)	0 (0, 1)	3 (0.25, 6)	< 0.001	85473
Positive rate of lymph nodes, Median (Q1,Q3)	0 (0, 0.46)	0 (0, 0.4)	0.55 (0, 0.83)	< 0.001	97059
Tumor status, n (%)				0.003	Fisher
Single send	2233 (88.9)	2115 (89.4)	118 (80.8)		
Multiple occurrences	234 (9.3)	208 (8.8)	26 (17.8)		
Multiple stoves	45 (1.8)	43 (1.8)	2 (1.4)		
Surgical method, n (%)				0.69	0.742
Unilateral resection	628 (25)	591 (25)	37 (25.3)		
Unilateral+ismus resection	962 (38.3)	902 (38.1)	60 (41.1)		
Unilateral+ismus+resection resection	922 (36.7)	873 (36.9)	49 (33.6)		
hashimotos, n (%)				0.343	0.9
no	2093 (83.3)	1976 (83.5)	117 (80.1)		
yes	419 (16.7)	390 (16.5)	29 (19.9)		
preop_tgab, n (%)				0.098	2.731
Normal	2303 (91.7)	2175 (91.9)	128 (87.7)		
Higher than normal value	209 (8.3)	191 (8.1)	18 (12.3)		
preop_tsh, n (%)				0.082	Fisher
Normal	2322 (92.4)	2193 (92.7)	129 (88.4)		
Higher than normal value	123 (4.9)	110 (4.6)	13 (8.9)		
Lower than normal value	67 (2.7)	63 (2.7)	4 (2.7)		
preop_sch, n (%)				0.032	4.605
Normal	2390 (95.1)	2257 (95.4)	133 (91.1)		
Higher than normal value	122 (4.9)	109 (4.6)	13 (8.9)		

The “statistic” column represents the test values for group comparisons.X² values (from chi-square test) or Fisher’s exact test values were used for categorical variables (e.g., sex, cancer type, and lnm_status). For continuous variables (e.g., age, tumor_max_diameter_cm, and lnm_count), the values represent t (from independent-sample t-test) orW/U statistics.

Significant intergroup differences were observed in both the training and validation cohorts with respect to maximum tumor diameter, number of metastatic lymph nodes, lymph node positivity rate, and age (all p < 0.05) (17). In contrast, no significant differences were identified for the remaining variables (p ≥ 0.05). Furthermore, no statistically significant differences in overall clinical characteristics were observed between the training and validation cohorts (p ≥ 0.05), suggesting a high degree of comparability between the two datasets.

### 3.2 Feature selection

**Figure 3:**
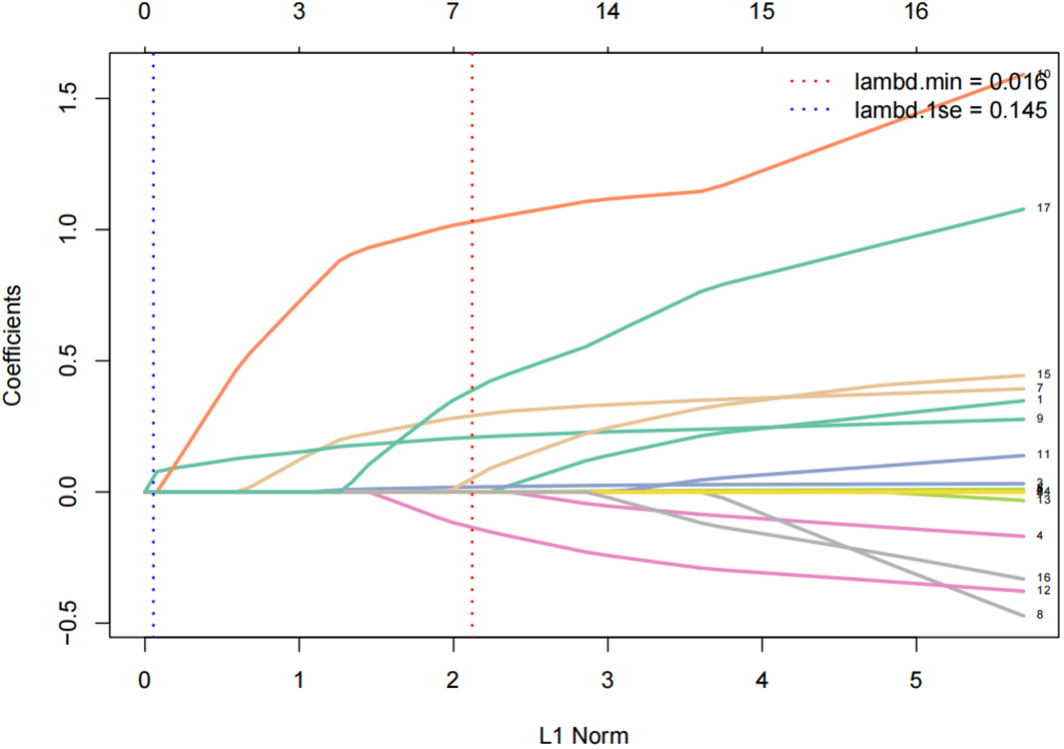
Feature selection using LASSO regression. Based on 10-fold cross-validation and the one-standard-error rule (lambda.1se), four independent predictors associated with recurrence were selected.

To reduce dimensionality and prevent overfitting, LASSO regression was used for feature selection. LASSO regression selected features with the lambda parameter set via 10-fold cross-validation, using the one-standard-error criterion for a simpler model (**Figure 3**). The four independent predictors identified by LASSO were: maximum tumor diameter, number of metastatic lymph nodes, lymph node ratio, and age. A concise model with four independent predictors—maximum tumor diameter, number of metastatic lymph nodes, lymph node ratio, and age—was developed using logistic regression for clinical prediction.

### 3.3 Model construction and evaluation

**Figure 4:**
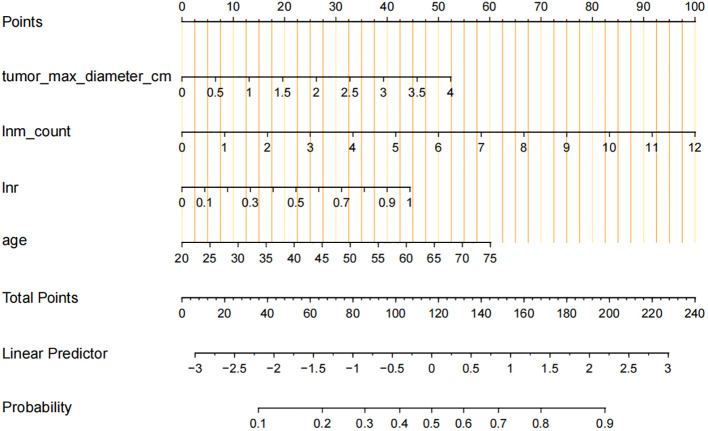
Nomogram for predicting recurrence in residual thyroid tissue. Constructed based on the multivariable logistic regression model, incorporating four predictors: maximum tumor diameter, number of metastatic lymph nodes, lymph node ratio, and age. Each predictor corresponds to a point scale at the top; the total points are summed and projected to the probability scale to estimate individualized recurrence risk.

**Figure 5:**
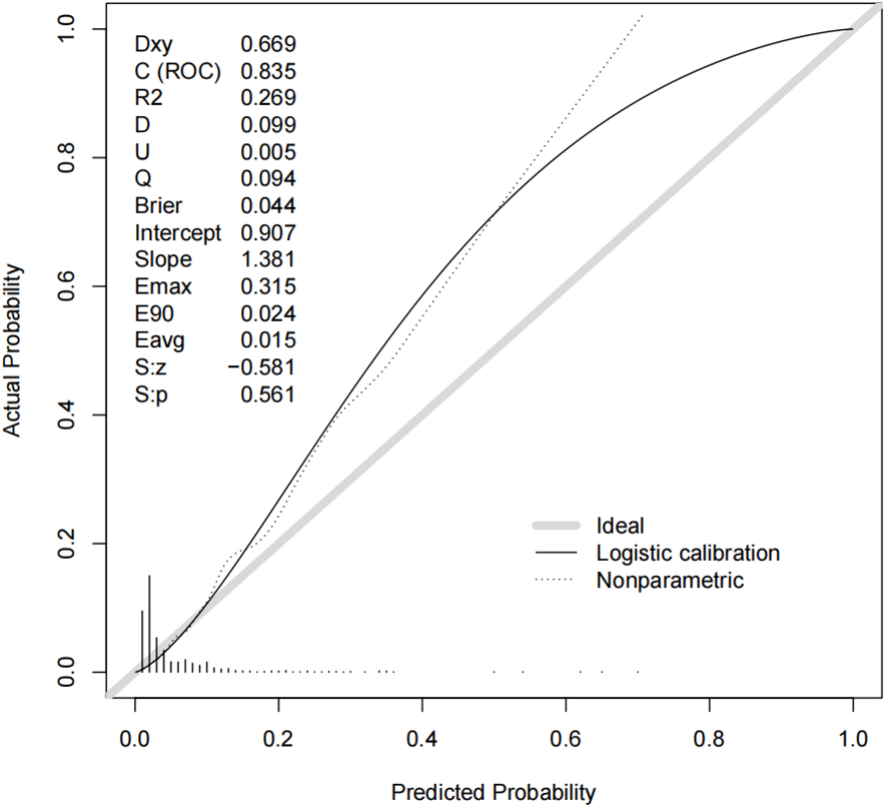
Calibration curves of the nomogram. The plots show the agreement between predicted probabilities and actual recurrence probabilities, with Brier scores of 0.115 and 0.142 in the training and validation sets, respectively, indicating good calibration of the model.

A nomogram was developed utilizing a multivariable logistic regression model that integrated the four previously mentioned predictors (**Figure 4**). The model exhibited strong discriminatory performance in both the training and internal validation datasets, with an Area Under the Curve (AUC) and 95% confidence intervals (CI) of 0.7518 and 0.8347, respectively (**Figure 1**, **5**). The optimal classification threshold was determined to be 0.245 based on the Youden index.

Constructed based on the multivariable logistic regression model, incorporating four predictors: maximum tumor diameter, number of metastatic lymph nodes, lymph node ratio, and age. Each predictor corresponds to a point scale at the top; the total points are summed and projected to the probability scale to estimate individualized recurrence risk).

### 3.4 Calibration & clinical utility

**Figure 6:**
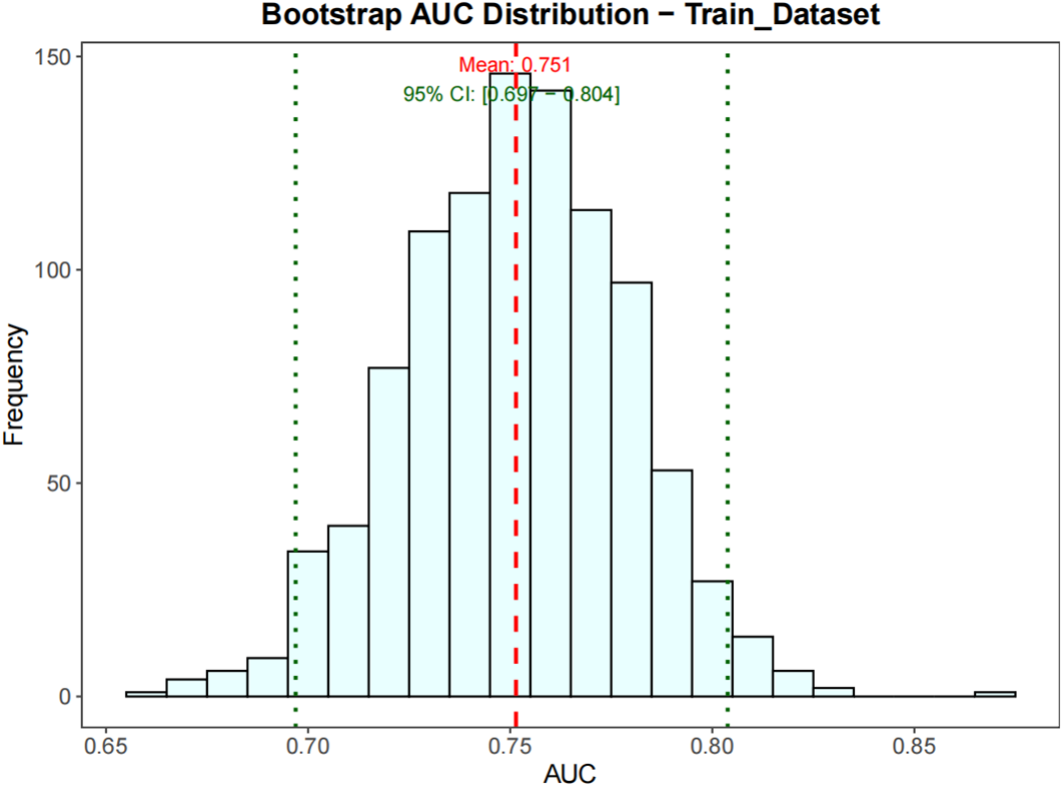
AUC distribution from Bootstrap resampling. The stability of the model’s discriminatory performance was evaluated using 1,000 Bootstrap resamples, with AUC values concentrated in the 0.70-0.75 range, confirming the robust discriminative ability of the model.

**Figure 6** shows the calibration curves for the nomogram based on multivariable logistic regression for both the training and internal validation sets, with Brier scores of 0.115 and 0.142. In the training set, sensitivity was 0.4757, specificity 0.8823, PPV 0.2008, and NPV 0.9644. The calibration analysis revealed a calibration intercept of 0.260 and a slope of 0.907, indicating good alignment between predicted probabilities and actual risks.

**Figure 7:**
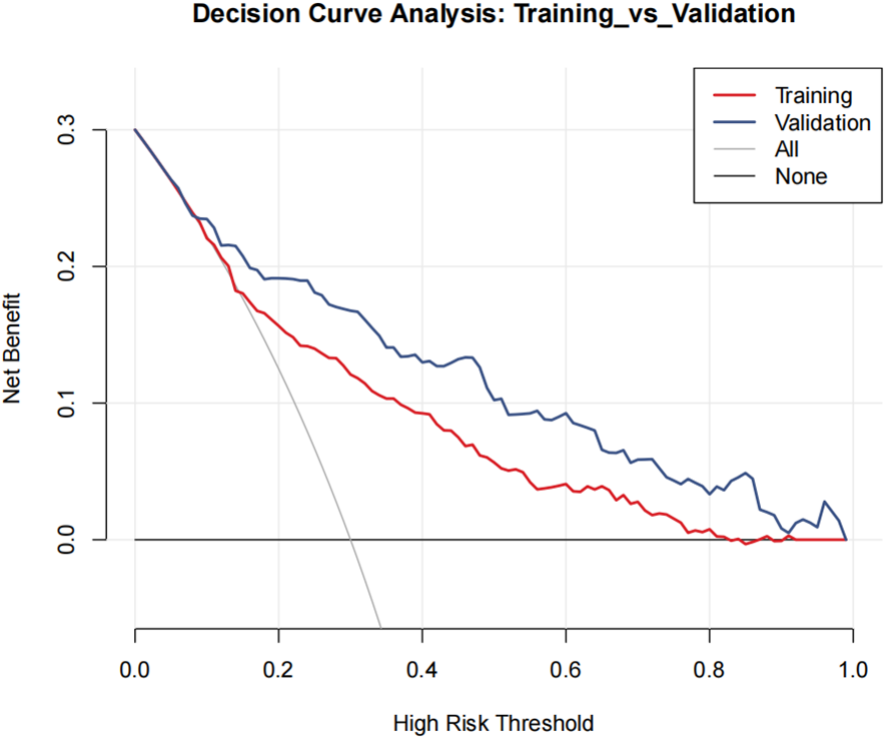
Decision curve analysis. Evaluation of the clinical net benefit of the nomogram in the training and validation sets. Within the high-risk threshold range of 0.1–0.5, the model (red and blue lines) demonstrated higher net benefit compared to both the “treat-all” (gray line) and “treat-none” (black line) strategies, confirming its good clinical utility.

**Figure 8:**
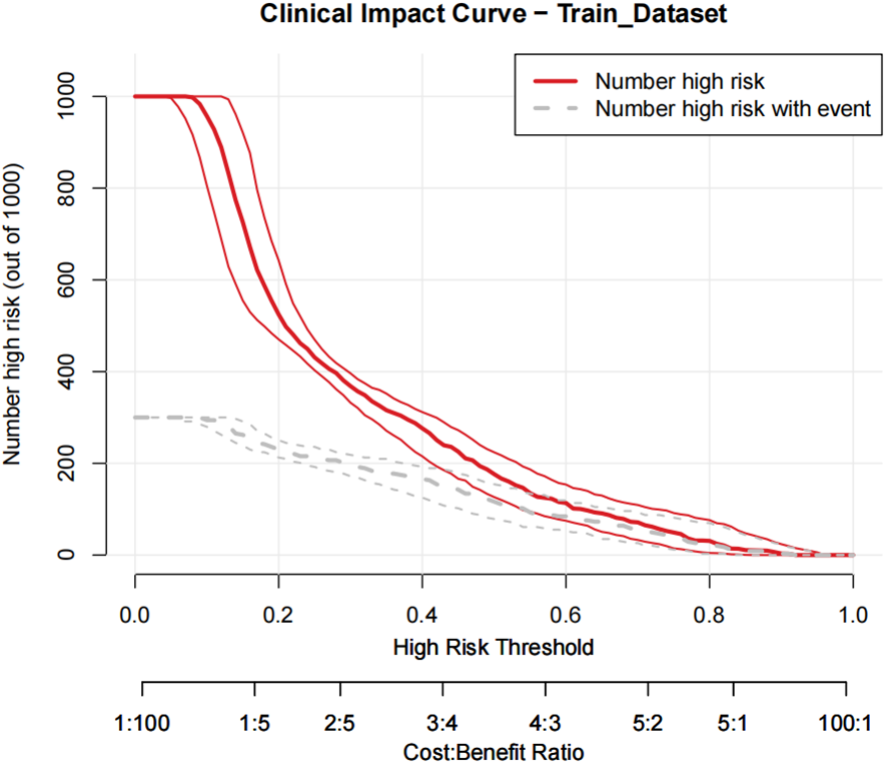
Clinical impact curve. The plot shows the total number of patients classified as high-risk by the model (blue line) and the number of those who actually experienced recurrence (red line) across different high-risk thresholds, intuitively reflecting the clinical identification efficacy of the model.

Decision curve analysis (DCA) demonstrated that, within the clinical decision threshold range of 0.1 to 0.5, the nomogram model yielded a superior net clinical benefit relative to both the “treat-all” and “treat-none” strategies, thereby affirming its substantial clinical utility (**Figures 7**, **8**).

### 3.5 Model explanation

The nomogram, developed from a multivariable logistic regression model, allocates specific points to each predictor variable. The cumulative score derived from these individual variables is used to compute a total score, which correlates with the probability of recurrence in the residual thyroid, as depicted in **Figure 1**. To expedite clinical evaluation, the **Supplementary Material** includes the full predictive equation, a sample scoring table, and detailed calculation steps. These resources empower clinicians to efficiently estimate personalized recurrence risk during bedside consultations or in outpatient environments.

## 4 Discussion

It is noteworthy that a discrepancy in AUC values was identified between the training set (0.7518) and the internal validation set (0.8347).DeLong’s test confirmed the statistical significance of this AUC difference (p = 0.0609). Importantly, no data leakage occurred, as all preprocessing steps were confined to the training set. The observed inversion is attributable to the ROSE-based balancing of the training set, which intentionally shifts the class distribution and makes the training task more challenging, leading to a conservative AUC estimate. In contrast, the validation set preserves the original disease prevalence and thus provides a more realistic assessment of model performance in clinical practice.

This phenomenon is primarily attributed to the application of undersampling techniques during the training phase to address class imbalance. Although undersampling improves the model’s capacity to detect the minority class (recurrent cases), it modifies the original distribution of the training data, which may result in an underestimation of the area under the curve (AUC) for that specific dataset. Conversely, the internal validation set preserved the natural distribution of the original data, thereby yielding evaluation results that more accurately represent the model’s generalization performance in real-world patient populations. Consequently, the higher AUC observed in the validation set compared to the training set suggests that, following optimization through undersampling, the model exhibits enhanced discriminatory power in independent samples reflective of the general population. Additionally, the randomness in data partitioning, coupled with effective overfitting control through regularization techniques such as LASSO and stepwise regression, contributed to the model’s sustained performance during the validation phase.

Our approach, using LASSO and stepwise regression, integrates multiple predictors like tumor size, lymph node ratio, and age, enhancing individualized probability prediction for residual thyroid recurrence when used alongside the traditional ATA staging system. Decision curve analysis indicates this model’s potential to aid in developing postoperative therapies and personalized follow-up plans, offering clinicians a straightforward and reliable decision-support tool (18).

Unlike earlier studies that used traditional ATA staging or single clinical factors for risk assessment, we improved our model’s robustness and clinical feasibility by employing a structured variable selection strategy (LASSO with stepwise regression), multiple imputation for missing data, and Bootstrap internal validation (3). Traditional staging models for predicting thyroid cancer recurrence risk usually show AUCs of 0.65 to 0.75. Our nomogram achieved an AUC of 0.835 in internal validation, and decision curve analysis indicated favorable net clinical benefit across thresholds of 10% to 50%. These findings suggest that the nomogram may add value to the current ATA framework by providing a more granular, individualized probability for residual thyroid recurrence.

Several recent studies have developed PTC recurrence prediction models, including application of five ML algorithms, construction of an ultrasound-based radiomics nomogram, and development of a multimodal deep learning model. Compared with these approaches, our nomogram offers several practical advantages. First, it uses only four routine variables readily available from standard pathology reports, whereas ML models often require extensive feature sets or specialized radiomics data. Second, it specifically targets ‘recurrence in the residual thyroid’—a distinct endpoint in patients with less-than-total thyroidectomy—rather than overall structural recurrence. Third, the nomogram format provides an intuitive, point-based tool for bedside risk estimation without specialized software. Our model achieved an AUC of 0.835 in internal validation, comparable to or better than some ML models despite using far fewer variables. We acknowledge that ML approaches may better capture complex non-linear interactions; however, their ‘black-box’ nature and data requirements may limit clinical adoption. Our nomogram represents a practical, interpretable alternative that balances performance with usability. Future external validation and direct comparisons with ML methods are warranted.

Analysis showed that the maximum tumor diameter, number of metastatic lymph nodes, and lymph node ratio were key predictors of thyroid recurrence. These results align with known pathology and clinical experience in papillary thyroid carcinoma, where tumor size indicates primary burden, and lymph node involvement reflects local spread and metastasis potential (10, 19).

Aligning with evidence-based medical consensus boosts the model’s credibility and clinical acceptance. The nomogram’s visual scoring system simplifies a complex multivariable model into an easy-to-use point-based format, allowing clinicians to quickly calculate a patient’s recurrence probability by summing indicator scores. This clear decision-making process reduces “black box” concerns, making it an objective and intuitive tool for physician-patient communication and shared decision-making.

Utilizing the nomogram model developed in this study, quantitative evidence can be furnished at the pivotal clinical juncture subsequent to the completion of postoperative pathological reports, thereby informing decisions regarding adjuvant therapy and personalized follow-up strategies. By employing risk stratification based on the probability threshold (0.245) established by the Our model provides an actionable risk probability. Patients with a predicted probability above the cut-off can receive intensified neck ultrasound surveillance or early re-intervention (e.g., completion thyroidectomy), whereas those below can follow standard protocols without undue anxiety or invasive procedures.index, clinicians are enabled to promptly identify and manage high-risk patients intensively, while simultaneously preventing excessive intervention in low-risk individuals, all within the confines of existing follow-up resource limitations. For cases with intermediate features or controversial treatment decisions, this model’s quantitative risk probability complements the ATA staging system by supporting decision-making for intermediate-risk patients, allowing finer risk differentiation, and providing a tool for dynamic assessment during consultations.

The model can be effectively converted into a clinical tool for real-time risk assessment and documentation in outpatient settings. It can be integrated into decision-support modules like mobile apps, web calculators, or HIS plugins. Physicians can input key pathological indicators, and the system quickly calculates scores and provides personalized recurrence probabilities and risk stratifications. The results can be automatically saved to electronic medical records, aiding standardized treatment protocols and long-term quality control. Stratified application for specific clinical subgroups, like age cohorts or tumor subtypes, may enhance follow-up efficiency and resource allocation. However, its effect on long-term disease-free survival and resource distribution needs validation through multicenter, prospective studies.

This study emphasizes scientific rigor and clinical applicability throughout its design, methodology, and practical implementation, with the objective of developing a predictive tool that is robust, transparent, and actionable.

The study employed a single-center consecutive enrollment strategy with a strict 7:3 temporal split for training and internal validation sets. Additionally, Bootstrap resampling (1,000 iterations) was used to validate internal performance, improving model stability and verifiability in similar populations.

In terms of variables and methodology, the study systematically addressed the issue of missing values in real-world data by employing Multiple Imputation by Chained Equations (MICE) and effectively managed multicollinearity through the application of LASSO regression. This approach ensured the quality and independence of the input data (20). During the model development process, we rigorously followed the guidelines set forth by the Transparent Reporting of a multivariable prediction model for Individual Prognosis Or Diagnosis (TRIPOD) statement. In addition to optimizing discriminatory performance, as measured by the area under the receiver operating characteristic curve (AUC), considerable attention was devoted to probability calibration using the calibrate function, and to assessing clinical utility through net benefit analysis in Decision Curve Analysis. This approach was undertaken to ensure that the model outputs are closely aligned with the practical requirements of clinical decision-making.

The final model uses just four common clinicopathological variables, simplifying clinical application. All analysis code, data processing steps, and model objects are publicly accessible in the **Supplementary Material**, promoting transparency, reproducibility, and supporting future validation, tool deployment, and continuous learning.

This study, despite its strengths, has limitations. It used a single-center enrollment strategy, which could introduce selection bias, even with a large sample size. Although internal validation was thorough, the model’s generalizability to other regions or healthcare systems needs testing with multicenter, prospective cohorts. Additionally, the model’s reference standard, based on recurrence events during clinical follow-up, may be affected by differences in follow-up intervals and diagnostic methods. To reduce bias, we used a standardized follow-up protocol and consistent endpoint definitions. Our model relies mainly on routine clinical and pathological data, which might limit its predictive power in atypical cases due to the absence of radiomics data. Additionally, variations in pathological workflows, imaging equipment, and information systems across centers could impact the model’s applicability. We recommend local data-driven adjustments or retraining for better adaptation during broader implementation (21).

## 5 Conclusion

In summary, this study successfully developed and validated a nomogram model utilizing preoperative and clinicopathological characteristics. The model, which is based exclusively on routine data, exhibits strong discriminatory and calibration capabilities for predicting the risk of recurrence in the residual thyroid following surgery in patients with papillary thyroid carcinoma. When used as an adjunct to the traditional American Thyroid Association (ATA) staging system, this model provides an individualized probability estimate for residual thyroid recurrence, offering clinicians an intuitive and practical quantitative tool for personalized postoperative risk stratification. It may complement the ATA framework by refining risk discrimination in patients with less-than-total thyroidectomy, particularly for the distinct event of residual thyroid recurrence (5).

## Data Availability

The original contributions presented in the study are included in the article/**Supplementary Material**. Further inquiries can be directed to the corresponding author.

## References

[1] Wang Y Wang L . Age- and sex-specific impact on the progression of low-risk papillary thyroid carcinoma under active surveillance: a meta-analysis. Front Oncol. (2025) 15:1547345. doi: 10.3389/fonc.2025.1547345 40606968 PMC12213731

[2] Volpi EM Ramirez-Ortega MC Carrillo JF . Editorial: recent advances in papillary thyroid carcinoma: progression, treatment and survival predictors. Front Endocrinol. (2023) 14:1163309. doi: 10.3389/fendo.2023.1163309 36960399 PMC10028267

[3] De Leo S Brigante G D'Elia S Censi S Madeo B Morelli S . Prospective validation of ata risk score for papillary thyroid microcarcinoma: an itco real-world study. J Clin Endocrinol Metab. (2025) 110:e4196–e204. doi: 10.1210/clinem/dgaf190 40165363 PMC12623051

[4] Valerio L Dalmiglio C Maino F Mattii E Trimarchi A Cartocci A . Dynamic risk stratification integrated with ata risk system for predicting long-term outcome in papillary thyroid cancer. Cancers (Basel). (2023) 15:4656. doi: 10.3390/cancers15184656 37760625 PMC10526505

[5] Croce L Teliti M Chytiris S Sparano C Coperchini F Villani L . The american thyroid association risk classification of papillary thyroid cancer according to presurgery cytology. Eur J Endocrinol. (2024) 190:165–72. doi: 10.1093/ejendo/lvae012 38298148

[6] Ullmann TM Papaleontiou M Sosa JA . Current controversies in low-risk differentiated thyroid cancer: reducing overtreatment in an era of overdiagnosis. J Clin Endocrinol Metab. (2023) 108:271–80. doi: 10.1210/clinem/dgac646 36327392 PMC10091361

[7] Castro MR Morris JC Ryder M Brito JP Hay ID . Most patients with a small papillary thyroid carcinoma enjoy an excellent prognosis and may be managed with minimally invasive therapy or active surveillance. Cancer. (2015) 121:3364–5. doi: 10.1002/cncr.29468 26079806

[8] Debnam JM Guha-Thakurta N Sun J Wei W Zafereo ME Cabanillas ME . Distinguishing recurrent thyroid cancer from residual nonmalignant thyroid tissue using multiphasic multidetector ct. AJNR Am J Neuroradiol. (2020) 41:844–51. doi: 10.3174/ajnr.A6519 32327435 PMC7228186

[9] Mezosi E . Editorial: predictors for aggressiveness of papillary thyroid carcinoma. Front Endocrinol. (2024) 15:1363836. doi: 10.3389/fendo.2024.1363836 38304465 PMC10831484

[10] Gao X Luo W He L Cheng J Yang L . Predictors and a prediction model for central cervical lymph node metastasis in papillary thyroid carcinoma (cn0). Front Endocrinol. (2021) 12:789310. doi: 10.3389/fendo.2021.789310 35154002 PMC8828537

[11] Xu S Huang H Zhang X Huang Y Guan B Qian J . Predictive value of serum thyroglobulin for structural recurrence following lobectomy for papillary thyroid carcinoma. Thyroid. (2021) 31:1391–9. doi: 10.1089/thy.2021.0209 34340593

[12] Nieto HR Thornton CEM Brookes K Nobre de Menezes A Fletcher A Alshahrani M . Recurrence of papillary thyroid cancer: a systematic appraisal of risk factors. J Clin Endocrinol Metab. (2022) 107:1392–406. doi: 10.1210/clinem/dgab836 34791326 PMC9016467

[13] Heng Y Feng S Yang Z Cai W Qiu W Tao L . Features of lymph node metastasis and structural recurrence in papillary thyroid carcinoma located in the upper portion of the thyroid: a retrospective cohort study. Front Endocrinol. (2021) 12:793997. doi: 10.3389/fendo.2021.793997 35145480 PMC8823057

[14] Cao D Xu N Chen Y Zhang H Li Y Yuan Z . Construction of a pearson- and mic-based co-expression network to identify potential cancer genes. Interdiscip Sci. (2022) 14:245–57. doi: 10.1007/s12539-021-00485-w 34694561

[15] Han H Yang L Jia W Chen X . Risk factors for predicting lateral lymph node metastasis of papillary thyroid carcinoma based on lasso-logistic regression. Front Endocrinol. (2025) 16:1642298. doi: 10.3389/fendo.2025.1642298 41059228 PMC12497606

[16] Chen C Guo Y Tian F Liu S Yang W Wang Z . A unified interactive model evaluation for classification, object detection, and instance segmentation in computer vision. IEEE Trans Vis Comput Graph. (2024) 30:76–86. doi: 10.1109/tvcg.2023.3326588 37883267

[17] Carrillo JF Volpi EM . Editorial: recent advances in papillary thyroid carcinoma: lymph node metastasis. Front Endocrinol. (2022) 13:1084034. doi: 10.3389/fendo.2022.1084034 36506079 PMC9731132

[18] Wang H Li Q Tian T Liu B Tian R . Improving the risk prediction of the 2015 ata recurrence risk stratification in papillary thyroid cancer. J Clin Endocrinol Metab. (2025) 110:534–41. doi: 10.1210/clinem/dgae465 38980946

[19] Metovic J Cabutti F Osella-Abate S Orlando G Tampieri C Napoli F . Clinical and pathological features and gene expression profiles of clinically aggressive papillary thyroid carcinomas. Endocr Pathol. (2023) 34:298–310. doi: 10.1007/s12022-023-09769-x 37208504 PMC10511602

[20] Deng K Luo Y Zuo H Chen Y Gu L Liu M . Self-supervised upsampling for reconstructions with generalized enhancement in photoacoustic computed tomography. IEEE Trans Med Imaging. (2025) 44:5117–27. doi: 10.1109/tmi.2025.3588789 40658576

[21] Yu J Cui Y Fu C Ma X Si C Huang Y . Comparison of ultrasound risk stratification systems for pediatric thyroid nodules. Front Endocrinol. (2024) 15:1350123. doi: 10.3389/fendo.2024.1350123 38572472 PMC10989271

