# Supplementary material for: Internal validation of a residual thyroid recurrence model after PTC
Source: Front Oncol. 2026 Jul 31;16:1827099. doi: 10.3389/fonc.2026.1827099 (PMC13472921; doi:10.3389/fonc.2026.1827099)
Supplement: Supplementary file 1 [file DataSheet1.docx]

## Supplementary Table S1 List of candidate predictor variables

| **Variable name** | **Variable type** | **Definition / Measurement** |
| --- | --- | --- |

| sex | Binary | female / male |
| --- | --- | --- |
| age, years | Continuous | age at surgery (years) |
| Tumor type | Binary | micro papillary carcinoma (≤1 cm) / papillary carcinoma (>1 cm) |
| tumor_max_diameter_cm | Continuous | maximum tumor diameter (cm) |
| Lymph node status | Binary | negative / positive |
| The number of lymph nodes | Continuous | number of metastatic lymph nodes (count) |
| Positive rate of lymph nodes | Continuous | positive nodes / total examined nodes |
| Tumor status | Ordinal | single focus / 2 foci / ≥3 foci |
| Surgical method | Nominal | unilateral resection / unilateral+isthmus resection / unilateral+isthmus+contralateral partial resection |
| hashimotos | Binary | no / yes (pathological diagnosis) |
| preop_tgab | Binary | normal / higher than normal value |
| preop_tsh | Ordinal | normal / higher than normal / lower than normal |
| preop_sch | Binary | normal / higher than normal value |

**Note:**

This table lists all candidate predictor variables considered before model development. Variable names are kept identical to those in Table 1 to ensure traceability. Variable types and measurement definitions are based on the data collection protocol of this study.

### Supplementary Table S2. Missing data summary for candidate variables

| **Variable** | **Total (N)** | **Missing (N)** | **Missing (%)** | **Variable type** |
| --- | --- | --- | --- | --- |

| lymph node positivity rate | 2512 | 506 | 20.14% | Continuous |
| --- | --- | --- | --- | --- |
| number of metastatic lymph nodes | 2512 | 495 | 19.71% | Continuous |
| lymph node status | 2512 | 438 | 17.44% | Categorical |
| recurrence status | 2512 | 0 | 0% | Categorical |
| sex | 2512 | 0 | 0% | Categorical |
| age | 2512 | 0 | 0% | Continuous |
| cancer_type | 2512 | 0 | 0% | Categorical |
| cancer_type_code | 2512 | 0 | 0% | Categorical |
| tumor_max_diameter_cm | 2512 | 0 | 0% | Continuous |
| tumor_foci | 2512 | 0 | 0% | Categorical |
| surgery_code | 2512 | 0 | 0% | Categorical |
| hashimotos | 2512 | 0 | 0% | Categorical |
| preop_tgab_cat | 2512 | 0 | 0% | Categorical |
| preop_tsh_cat | 2512 | 0 | 0% | Categorical |
| preop_sch | 2512 | 0 | 0% | Categorical |

Note: Missing data were handled using MICE multiple imputation (20 iterations, m=10). Trace plots indicated good convergence.
